# Three-dimensional analysis of human pancreatic cancer specimens by phase-contrast based X-ray tomography – the next dimension of diagnosis

**DOI:** 10.1186/s40644-023-00559-6

**Published:** 2023-05-02

**Authors:** Diana Pinkert-Leetsch, Jasper Frohn, Philipp Ströbel, Frauke Alves, Tim Salditt, Jeannine Missbach-Guentner

**Affiliations:** 1 Department of Diagnostic and Interventional Radiology, University Medical Center, Goettingen, Germany; 2 Institute for X-ray Physics, Georg-August-University, Goettingen, Germany; 3 Department of Pathology, University Medical Center, Goettingen, Germany; 4 Cluster of Excellence “Multiscale Bioimaging: from Molecular Machines to Networks of Excitable Cells” (MBExC), University of Goettingen, Goettingen, Germany; 5 Department of Hematology and Medical Oncology, University Medical Center, Goettingen, Germany; 6 Translational Molecular Imaging, Max-Planck-Institute for Multidisciplinary Sciences, Goettingen, Germany

**Keywords:** Pancreatic cancer, Precursor lesion, Synchrotron, Phase-contrast, X-ray based tomography, Virtual histology

## Abstract

**Background:**

The worldwide increase of pancreatic ductal adenocarcinoma (PDAC), which still has one of the lowest survival rates, requires novel imaging tools to improve early detection and to refine diagnosis. Therefore, the aim of this study was to assess the feasibility of propagation-based phase-contrast X-ray computed tomography of already paraffin-embedded and unlabeled human pancreatic tumor tissue to achieve a detailed three-dimensional (3D) view of the tumor sample in its entirety.

**Methods:**

Punch biopsies of areas of particular interest were taken from paraffin blocks after initial histological analysis of hematoxylin and eosin stained tumor sections. To cover the entire 3.5 mm diameter of the punch biopsy, nine individual tomograms with overlapping regions were acquired in a synchrotron parallel beam configuration and stitched together after data reconstruction. Due to the intrinsic contrast based on electron density differences of tissue components and a voxel size of 1.3 μm achieved PDAC and its precursors were clearly identified.

**Results:**

Characteristic tissue structures for PDAC and its precursors, such as dilated pancreatic ducts, altered ductal epithelium, diffuse immune cell infiltrations, increased occurrence of tumor stroma and perineural invasion were clearly identified. Certain structures of interest were visualized in three dimensions throughout the tissue punch. Pancreatic duct ectasia of different caliber and atypical shape as well as perineural infiltration could be contiguously traced by viewing serial tomographic slices and by applying semi-automatic segmentation. Histological validation of corresponding sections confirmed the former identified PDAC features.

**Conclusion:**

In conclusion, virtual 3D histology via phase-contrast X-ray tomography visualizes diagnostically relevant tissue structures of PDAC in their entirety, preserving tissue integrity in label-free, paraffin embedded tissue biopsies. In the future, this will not only enable a more comprehensive diagnosis but also a possible identification of new 3D imaging tumor markers.

## Introduction

Pancreatic ductal adenocarcinoma (PDAC) is one of the most lethal cancer malignancies with a 5-year survival rate less than 10% with numbers that continue to rise steadily [1–3]. Of all pancreatic neoplasia PDAC is the most common type accounting for about 90% of pancreatic malignancies, mainly characterized by pronounced desmoplasia and morphologically altered duct stratification [2, 4]. The often atypical symptoms that appear very late in the PDAC disease progression and the non-existing screening options for pancreatic cancer and its precursor lesions make early detection almost impossible [5]. Thus, pancreatic cancer is usually detected much too late in accordance with the fact that the median survival after occurrence of metastasis or local invasion varies between six and twelve months [6]. Only an early and complete surgical resection provides chances for long-term survival. While excellent progress has been made in refining classifications of the genetic diversity of pancreatic cancers which also is reflected in various histopathological subclassifications of PDAC and its many precursor lesions, no major breakthrough in therapy development was made so far [7–11]. That is why PDAC as one of the most therapy resistant tumor entities is still treated mainly by neo-adjuvant and adjuvant chemotherapy [1, 12–14]. The emergence of PDAC can occur both via development through multiple precursors and independently of them [15]. The best-known precursor lesions for PDAC are pancreatic intraepithelial neoplasia (PanIN) and intraductal mucinous neoplasia (IPMN) [5, 11]. Both have pancreatic ductal cells as their neoplastic origin and are usually asymptomatic in their gradual development. Since precursor lesions and cancer cells occur at the same time, it is possible that preoperative punctures and punch biopsies for histological confirmation of the diagnosis are inadequately classified and that false negative findings are made on the basis of the selected tissue. Thus, the preoperative diagnosis of pancreatic cancer by standard histological procedure is sometimes difficult and inconclusive [10, 16]. In addition to the preoperative histological diagnosis, a high-quality histopathological assessment of the resected tumor tissue is also of particular clinical relevance to evaluate the success of the surgical resection. The margin status, which is represented by the R classification, plays a pivotal role in further therapeutical intervention and prognosis. Multiple tumor cell positive margins of the pancreatic resectate are a prognostic relevant parameter that influences the postoperative treatment and increase the risk of cancer recurrence and decreased survival after resection [17, 18]. The detection and histopathological diagnosis of PanIN is usually performed microscopically due to its small size. IPMN, on the other hand, is a cystic epithelial neoplasm that can be found in pancreatic ducts of various calibres. Asymptomatic, smaller cysts are sometimes detected as incidental findings on computed tomography (CT) of the abdomen [10, 19]. However, the detection of an IPMN as a significant precursor lesion of PDAC entails an unambiguous classification according to Fukuoka consensus guidelines, which underlines the special importance of imaging methods in PDAC and its precursor lesions [20, 21]. Thereby CT, magnetic resonance imaging or magnetic resonance cholangiopancreatography visualize among others the size of the cystic lesions and their location, the dilation of the main duct and other criteria, which define the classification of “worrisome features” or “high-risk stigmata”. The assessment of defined morphological features has direct consequences for the individual treatment and leads to either a spacious resection or a surveillance strategy. Accordingly, the search for prognostic factors that lead to an assessment of the individual patient’s disease course is a major demand. While R-status has been established as a prognostic marker for resections, it is still a controversial issue whether the occurrence of the precursor lesions PanIN and IPMN and their classification into high-risk and low-risk precursor within the PDAC tumor have an influence on overall survival after resection [21–24]. Up to now, the gold standard in the diagnosis of PDAC and its precursor lesions is still histopathological examination and evaluation in thin two-dimensional (2D) tissue sections, usually hematoxylin and eosin (H&E) stained. This always entails destruction of the tissue sample in the first step. Thereby, the histological sections represent only a fraction of the normally much larger tissue sample. The importance of three-dimensional (3D) visualization for obtaining complex information, particularly with respect to tumor biology, has been the subject of several studies, including PDAC and its predecessors (IPMN, PanIN), in which serial sections of tumor specimens were automatically stitched together after histological staining [25, 26]. They showed that the areas that appeared as individual tumor nests in 2D sections were part of the overall tumorous structure in the 3D image. The actual extent of a tumor is significantly underestimated by pure 2D analysis. Therefore, there is a high need to visualize tissue samples in form of a 3D virtual histology in a non-destructive manner. The ex vivo 3D analysis of tissues and organs using phase-contrast X-ray computed tomography (PCCT) has emerged as a new imaging tool for biomedical research in recent years for high resolution analysis, quantification of specific structures and identification of regions of interest, that otherwise would have been overlooked [27]. The challenge here is to visualize soft tissue, which is intrinsically low in contrast. For contrast enhancement special staining protocols, which for example contained phosphotungstic acid (PTA), were used. In combination with synchrotron radiation, not only morphological structures of embryos, lung, heart, kidney but also pathological alterations of vessels or kidneys were assessed in detail with excellent contrast and resolution [28–32]. In PTA contrasted pancreatic tissue, we previously showed that tumor and healthy pancreatic tissue can be delineated, based on PCCT reconstructions. Therefore, voxel sizes in between 167 and 658 nm using a multiscale implementation of PCCT were used. Furthermore, we could visualize Islets of Langerhans at single cell level in 3D and could quantify the presence of collagen fibers [33]. Although contrasting of soft tissue for x-ray based imaging works very well, additional staining has several disadvantages in terms of a longer time for sample preparation and altered histochemical staining pattern [32]. However, most importantly, this approach excludes analyses of already paraffin-embedded specimens. Recently, X-ray tomography was even realized label free in murine and human soft tissue samples. First by grating-based PCCT at anatomical resolution, and also at histological resolution upon continuously adapting configurations of the propagation-based X-ray phase-contrast tomography setups at the synchrotron [31, 34–39]. Here, we demonstrate that PCCT allows to track pathologically altered structures in unstained PDAC punch biopsies in 3D. This allows to assess their true extent across the entire volume. Moreover, these tissue samples can subsequently further be validated by histological and immunohistochemical procedures. Our work clearly shows that compared to planar 2D histological diagnosis alone, 3D X-ray based tomography of unstained paraffin embedded human PDAC tissue samples leads to more comprehensive information of the 3D tumor architecture. We anticipate that the additional 3D histological information about the cancer tissue will contribute to a more accurate diagnosis, possibly assessing R-status more comprehensively and thus directing patients to the best individualized treatment. Furthermore, the 3D imaging of tumor histologies has the potential to identify new, as yet undiscovered 3D cancer imaging biomarkers that have additional diagnostic and prognostic value for patients.

## Materials and methods

### Sample origin

Human specimens of pancreatic lesions from four different patients were obtained in the form of paraffin blocks from the Department of Pathology, University Medical Center (Goettingen, Germany). Pathological diagnosis was made as part of each patient’s treatment plan. The study was performed according to the guidelines of the local ethics committee of the University Medical Center Goettingen (Ethics Vote 24/4/04) and in accordance with the Declaration of Helsinki.

### Workflow and sample preparation

**Fig. 1:**
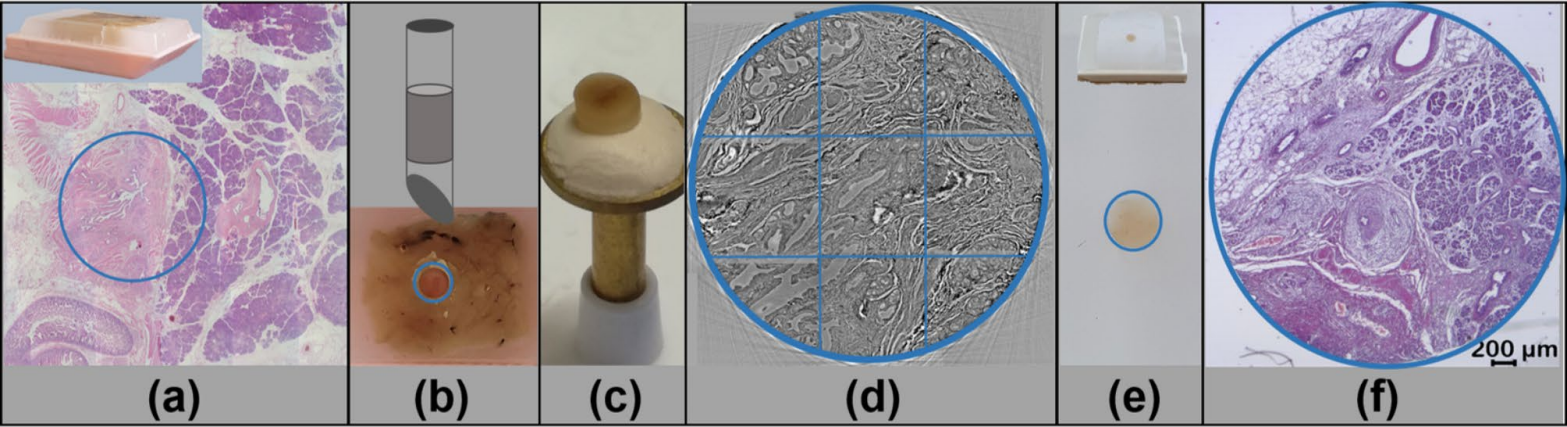
Schematic workflow. First, the area of interest was identified from H&E stained 2 μm tissue slices obtained from the patient paraffin tissue block (a, blue circle). A 3.5 mm punch biopsy was taken from the selected area (b). The paraffin tissue cylinder was removed from the punch and directly mounted on a brass pin (c). A representative 2D slice through the volume of the PCCT scan of the biopsy is shown, recorded at the P10/PETRA III synchrotron source (DESY, Hamburg, Germany). To this end, 9 individual tomograms with overlapping regions were stitched together subsequently (d, blue grid). Afterwards the punch biopsies were again embedded in paraffin (e). For correlative analysis, 2 μm paraffin sections were sliced and stained with H&E (f)

After initial histological evaluation of H&E stained 2 μm tissue sections of paraffin-embedded pancreatic cancer tissue samples areas of particular interest were selected. Thereafter, 3.5 mm diameter punch biopsies were taken from the paraffin block and mounted on brass pins as corresponding holder (Fig. 1a-c). X-ray phase-contrast tomography was performed by taking 9 individual tomograms per sample which were stitched together afterwards (Fig. 1d). Next, the biopsy was embedded in paraffin and was then sliced into 2 μm sections for further histological and immunohistochemical stainings (Fig. 1e, f).

### Synchrotron computed tomography set up

The measurements of the punch biopsies were performed at the GINIX endstation of the beamline P10/PETRA III at DESY (Hamburg, Germany). The energy of the parallel beam configuration was 13.8 keV with the propagation distance of 500 mm. 1500 tomographic projections per 180° angular interval with a pixel size of 650 nm and a detector field of view of 1.6 mm x 1.4 mm were acquired using a scintillator (50 μm LuAG:Ce) coupled X-ray microscopy (Optique Peter, France) equipped with a sCMOS camera (PCO edge, PCO, Germany). The exposure time per projection was 35 ms. To cover the entire 3.5 mm diameter punch biopsy, 3 × 3 individual tomograms with overlapping regions were taken (Fig. 1d).

### Data reconstruction and segmentation

To enhance the contrast of the low absorbing soft tissue paraffin embedded samples, X-ray phase-contrast was exploited using Paganin’s algorithm for phase retrieval from the MATLAB too [40, 41]. Based on the overlapping tomograms, the reconstruction volume amounts to ~ 4 mm x 4 mm x 1.4 mm. By reducing the data size through a factor 2 binning, the final reconstructed data had a voxel size of 1.3 μm.

Segmentation was performed semi-automatically by means of the machine learning software *Ilastik* [42]. First, the features of interest are marked manually. Afterwards an automatic segmentation is suggested for the entire 3D volume based on the segmented pixel. In an iterative manner the automatic segmentation is adjusted by marking additional pixel manually.

The final segmentation is visualized in the commercial software *avizo* (ThermoFisher Scientific, Waltham, USA).

### Histology

The X-ray based imaging results were validated by histological processing of the punches as follows. The paraffinized tissue punch was embedded in a paraffin block (Fig. 1e) and sliced into 2 μm tissue sections (Fig. 1f). For further staining the tissue slices were deparaffinized (60 °C, 30 min) and rehydrated by a descending ethanol series. Both the H&E stain and the Movat’s pentachrome stain were performed according to the manufacturer’s protocol [43]. Immunohistochemistry targeting the epithelial marker Cytokeratin 18 (CK-18) was performed by treating the rehydrated tissue slices as follows: target retrieval solution (pH 9, 100 °C, 20 min), H_2_O_2_ (10 min, room temperature), Seablock (20 min, room temperature), αCK-18 (mouse anti-human, clone DC10, (DAKO, Hamburg, Germany) 1:50, 4 °C, overnight), secondary antibody (anti mouse horseradish peroxidase (Histofine, Nichirei Biosciences, Japan); 30 min, room temperature), finally AEC (3-Amino-9-ethylcarbazole) substrate (20 min, room temperature). Washing steps in between were carried out with Tris-(2-Amino-2-(hydroxymethyl)-1,3-propandiol) buffer (2 × 5 min, room temperature). After each individual staining the tissue slices were covered with corresponding mounting medium and cover glass for evaluation by light microscopy.

## Results

### Characteristic features of pancreatic lesions, visible in virtual 2D slices of PCCT volume, are comparable to standard histology

**Fig. 2:**
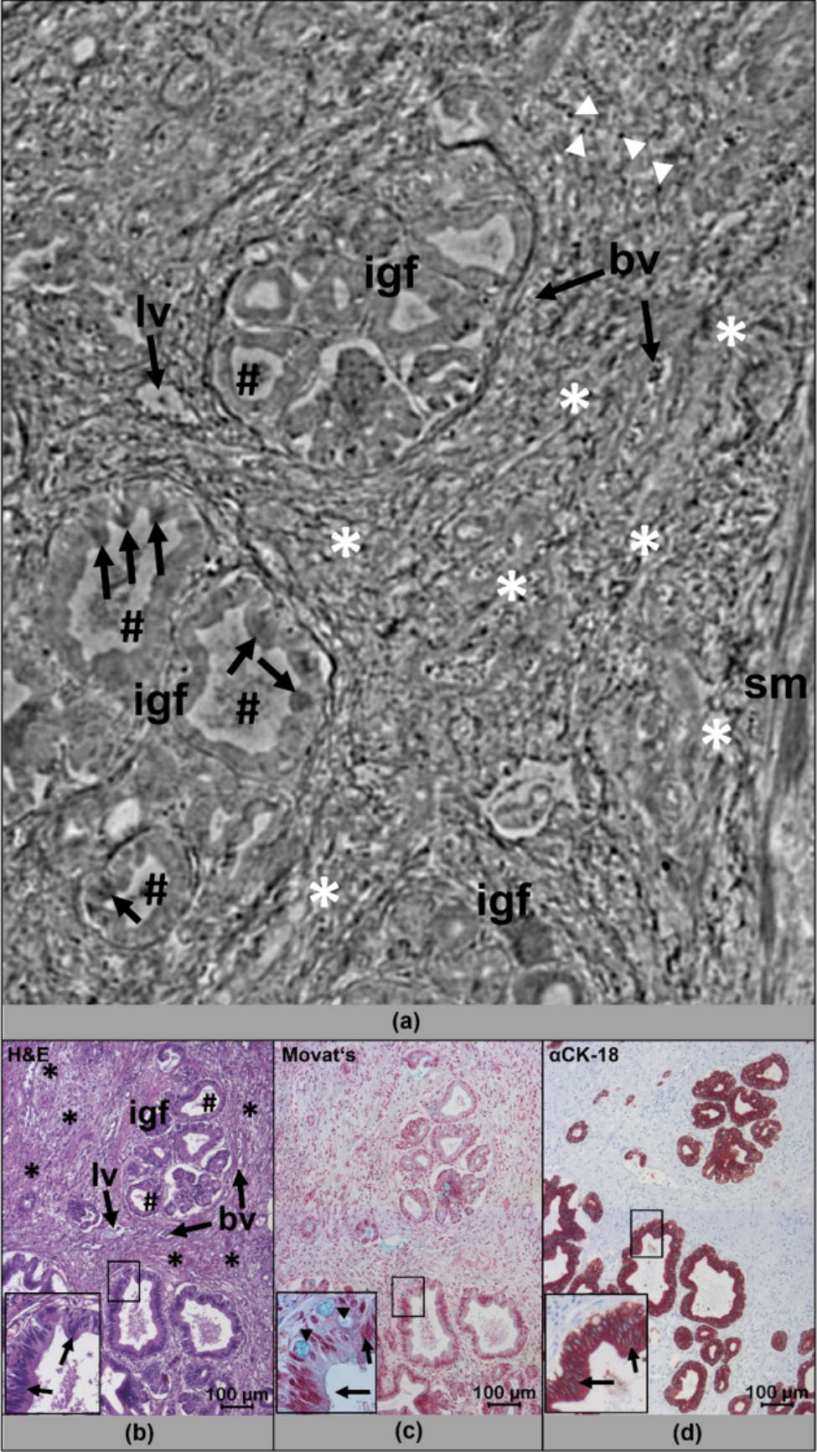
PCCT cross-sectional image of PDAC provides comparable information to conventional histology. A representative virtual 2D slice of the reconstructed 3D volume of a human PDAC punch biopsy (a) shows typical features of pancreatic cancer lesions: irregular small to medium size gland formations (igf) with intraductal epithelial proliferation (arrows). Note, that the accumulated cell nuclei can be identified due to their higher X-ray density. Most of the narrow duct lumina contain a mucous secret, which is clearly visible (hashtag). In addition, the desmoplastic stroma (white asterisks) shows infiltrations of dense lymphocytes (white arrow heads) as well as lymphatic vessels (lv) and blood vessels (bv), most likely veins. Smooth muscle fibres (sm) pervade the desmoplastic tissue and are clearly visible. H&E (b) and Movat’s pentachrome stain (c) of corresponding paraffin tissue sections of the punch biopsy confirmed the existence of the morphological structures visualized by virtual X-ray based histology in a). Note, that mucous secreting goblet cells as revealed by Movat’s pentachrome stain (arrow heads) are not detectable in the X-ray histological image. Antibody staining against CK-18 (d, red) marks cells of epithelial origin and depicts the multi-layered desmoplastic epithelium of the proliferating pancreatic glands (d, arrows). More details are presented from the magnification of a corresponding image section within b-d

To assess the diagnostic value of the phase-contrast X-ray tomography data from the punch biopsies of four human pancreatic cancer samples (workflow, Fig. 1), the virtual slices of the acquired 3D data sets were first examined for features of pathological alterations characteristic for PDAC. All tomograms, especially virtual sections that show typical signs of pancreatic precursor lesions were selected in a next step for a more detailed analysis. With this approach, regions of extensive stromal connective tissue accumulation, a hallmark of pancreatic cancer that had largely displaced the glandular pancreatic parenchyma, were identified in all data sets of the four PDAC biopsies (Fig. 2a). The used PDAC specimen was taken from a duodenopancreatectomy with R1-status. Intrinsic contrasting of the tissue and thus identification of these target structures was made possible by the fact that cell nuclei, especially heterochromatic cell nuclei, but also accumulations of extracellular matrix presented themselves with different electron density and corresponding phase shifts (Fig. 2a). Intraluminal structures consisting of cell debris and glandular mucous secretions also appeared quite dense (Fig. 2a). This natural tissue contrast allowed clear identification of individual glands and pancreatic ducts within the biopsies by X-ray phase-contrast imaging, that often appeared in irregular gland formations due to epithelial proliferation (Fig. 2a). After embedding the punch biopsies in paraffin blocks, tissue sectioning, histological / immunohistochemical analysis (Figs. 1e-f and 2b-d), and comparison with the PCCT X-ray based tomographic findings, we showed that both methods provided almost the same morphological information about the pathologic alterations of the pancreatic lesions e.g. irregular gland formations, connective tissue accumulation in terms of resolution and contrast (Fig. 2a-b). Goblet cells were overrepresented in the epithelia and thus judged as mucinous-differentiated glandular cell complexes which were also clearly seen after Movat’s pentachrome staining (Fig. 2c). Note, that the mucin-containing goblet cells were not detectable in the X-ray data sets. By staining with the epithelial specific antibody against CK-18 the epithelial cells of the proliferating pancreatic ducts and their multilayered structure were visualized (Fig. 2d).

### Tracking an altered pancreatic duct reveals in 3D the discontinuity of its morphology

**Fig. 3:**
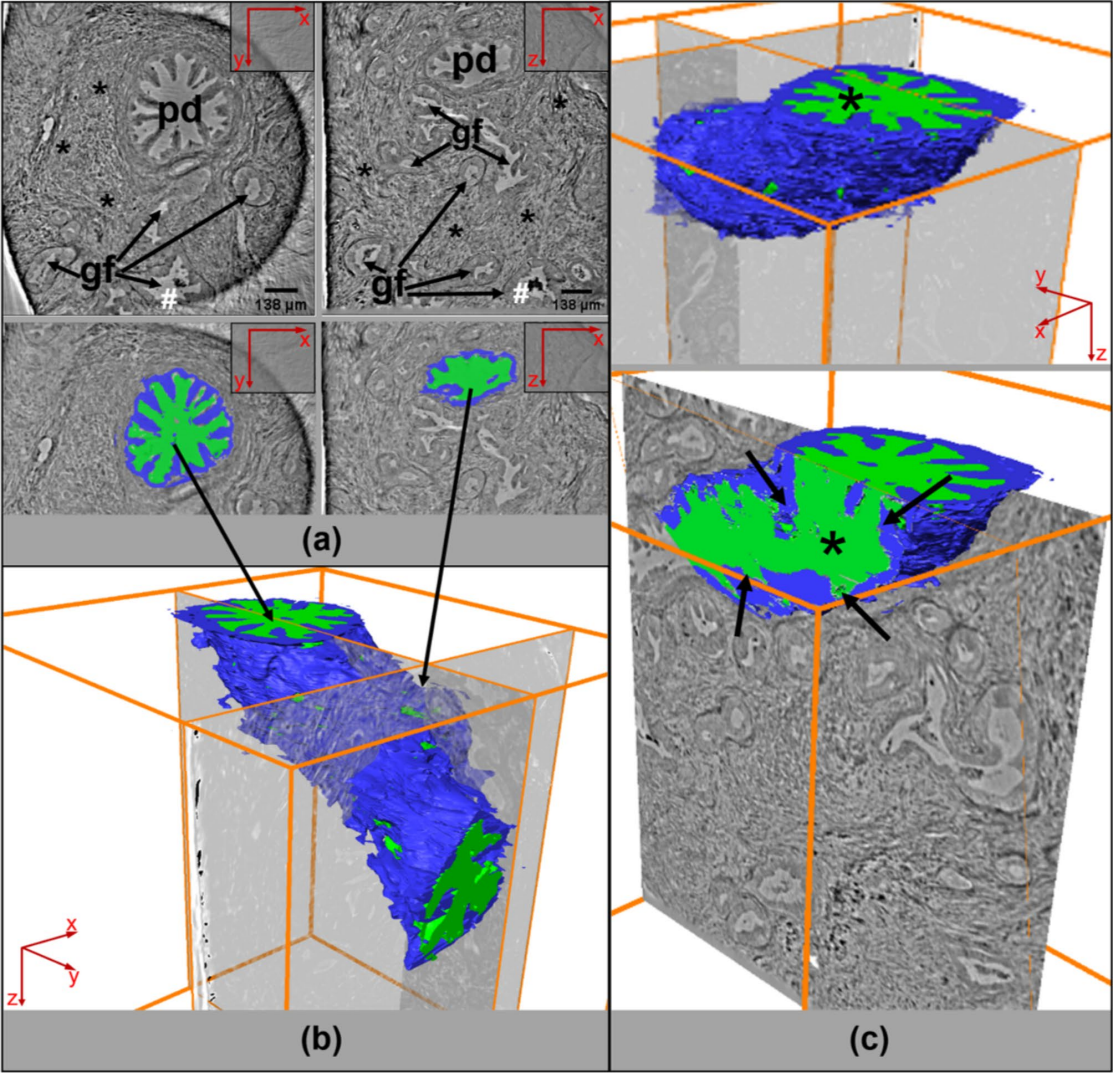
3D PCCT data set analysis of an altered pancreatic duct. Virtual sections from the X-ray tomogram of the biopsy punch from the virtual section number 42 (a, left) to a more distant virtual section number 760 (a, right) confirm the existence of altered gland formations (gf) in between the desmoplastic stroma (asterisks) as well as a star-like proliferating duct (pd) characterized by an altered epithelial morphology. Remarkably, very dense inclusions are observed within the lumina (hashtag). Red arrows in (a) indicate the displayed spatial direction of the virtual 2D tissue section. The star-like proliferating duct (pd) was semi-automatically segmented (b) through the whole volume, discriminating the lumen (green) and the epithelium (blue). The 3D reconstruction reveals the location of this duct within the biopsy volume from section 42 to the last traceable plane (b). The translucent layer marks the layer 760 in xz direction (b, right arrow). The angle of the segmented duct was virtually changed in order to evaluate a cross-section of the morphologically altered duct (c). Note, that the highly variable diameter of the lumen (c, star, green), caused mainly by the surrounding epithelium of varying height (arrows), indicates different epithelial proliferation rates in certain duct locations

By viewing serial virtual sections of 3D X-ray scans in these biopsies, not only typical PDAC features but also characteristics of other pancreatic lesions were detected within the data set and traced over the entire tissue punch. In a specimen of a duodenopancreatectomy with R1-status numerous altered pancreatic ducts were detected. The 3D reconstruction of one of these ducts revealed the actual dimensions within the biopsy punch. This way, the pancreatic duct ectasia of different caliber and atypical shape could be contiguously traced demonstrating a duct with irregular dilatations and numerous bifurcations (Fig. 3a). To visualize the lumen caliber of this altered duct in relation to its surrounding epithelial height across the entire data set, both the lumen of the altered pancreatic duct and its surrounding epithelium were segmented. Virtual slice planes through the segmented duct impressively showed a rapidly changing architecture with alternating dilated and compressed lumen areas caused by locally different epithelial proliferations within the same duct, forming, for example, a star shape (Fig. 3a-c). If individual 2D tomographic slices were viewed, a different classification and diagnosis resulted as shown in Fig. 3a depending on the orientation or imaging of the duct, compared to the duct morphology considered in its entirety in 3D, as shown here. The cross-section through the segmented duct shows the different distribution ratio of lumen and ductal epithelium which is also reflected in the virtual sections (Fig. 3a, c). This duct formation clearly correlates to a PanIN lesion.

### 3D analysis of IPMN ducts demonstrate more architectural information than conventional 2D pathohistology alone

**Fig. 4:**
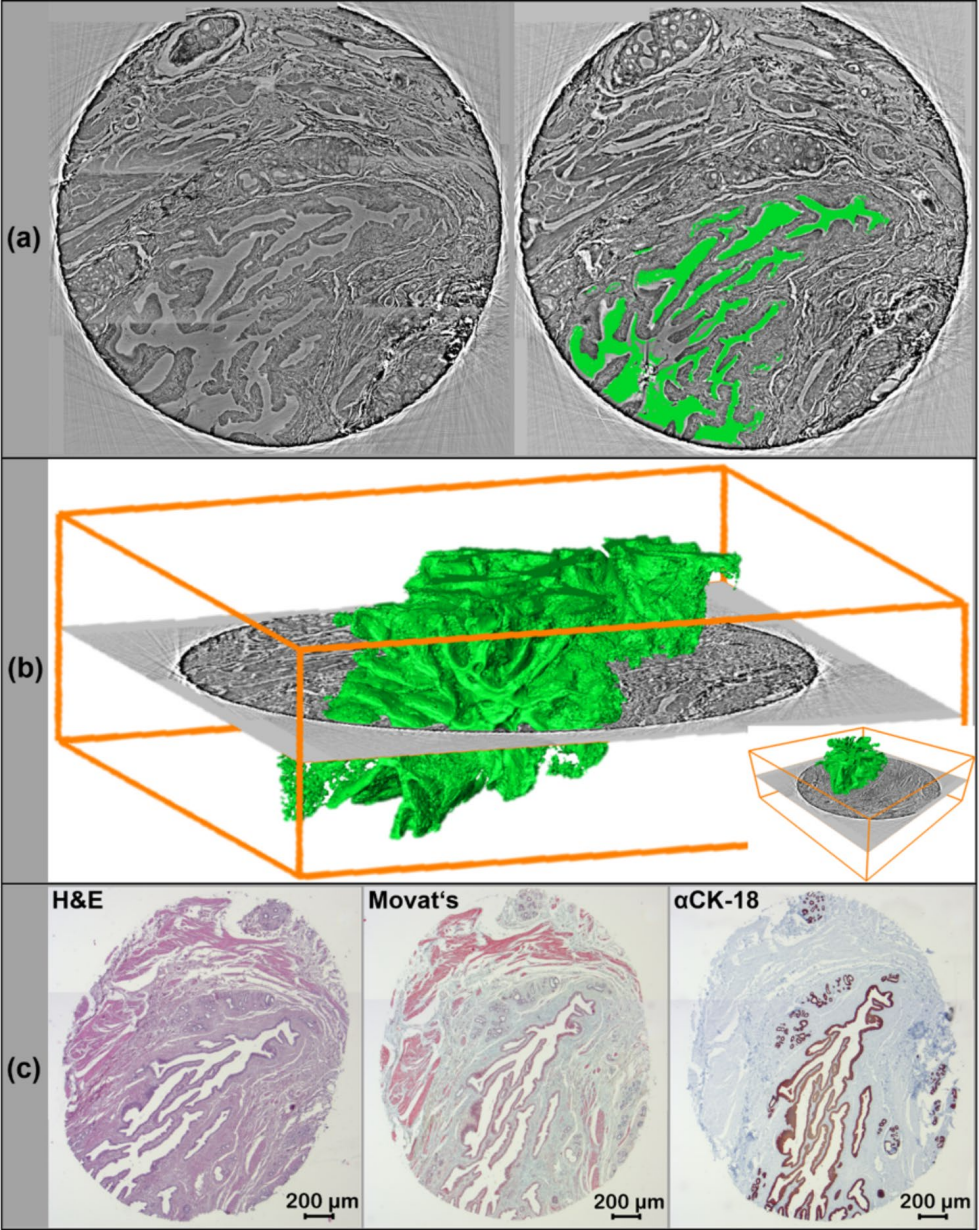
3D appearance of an IPNM with finger-like protrusions. A representative virtual 2D X-ray based slice of the punch biopsy is shown (a). Semi-automated segmentation of the lumen of the papillary duct structure in a virtual 2D section (a, right green) as well as over the entire punch biopsy volume is presented in the form of an endocast. (b, green). The corresponding H&E stained section demonstrated a general overview of the different tissue structures, Movat’s pentachrome stain visualized connective tissue, mucines, and muscle cells and specific antibody staining with anti CK-18 antibody displayed all epithelial cells (c)

Among other features, IPMN pancreatic lesions are characterized by cystically dilated pancreatic ducts. Therefore, a pancreatic biopsy (R0-status resected duodenopancreatectomy preparation) with prototypic IPMN on conventional histology was chosen for further 3D analysis (Fig. 4). The tomographic X-ray generated data set showed this IPMN typical ductal structure throughout the biopsy extending to approximately one third of the entire tissue. Based on the individual tomographic projections the lumen of the dilated duct was reconstructed and visualized in 3D (Fig. 4b). The obtained “cast” showed an extensive structure with multiple folded and long, finger-like protrusions. Although both the virtual 2D sections and the conventional 2D histology revealed numerous epithelial bridges and interruptions (Fig. 4a, c), the 3D impression of the dilated ductal system was able to demonstrate a coherent and complex structure (Fig. 4b).

### X-ray based analysis suggests perineural infiltration

**Fig. 5:**
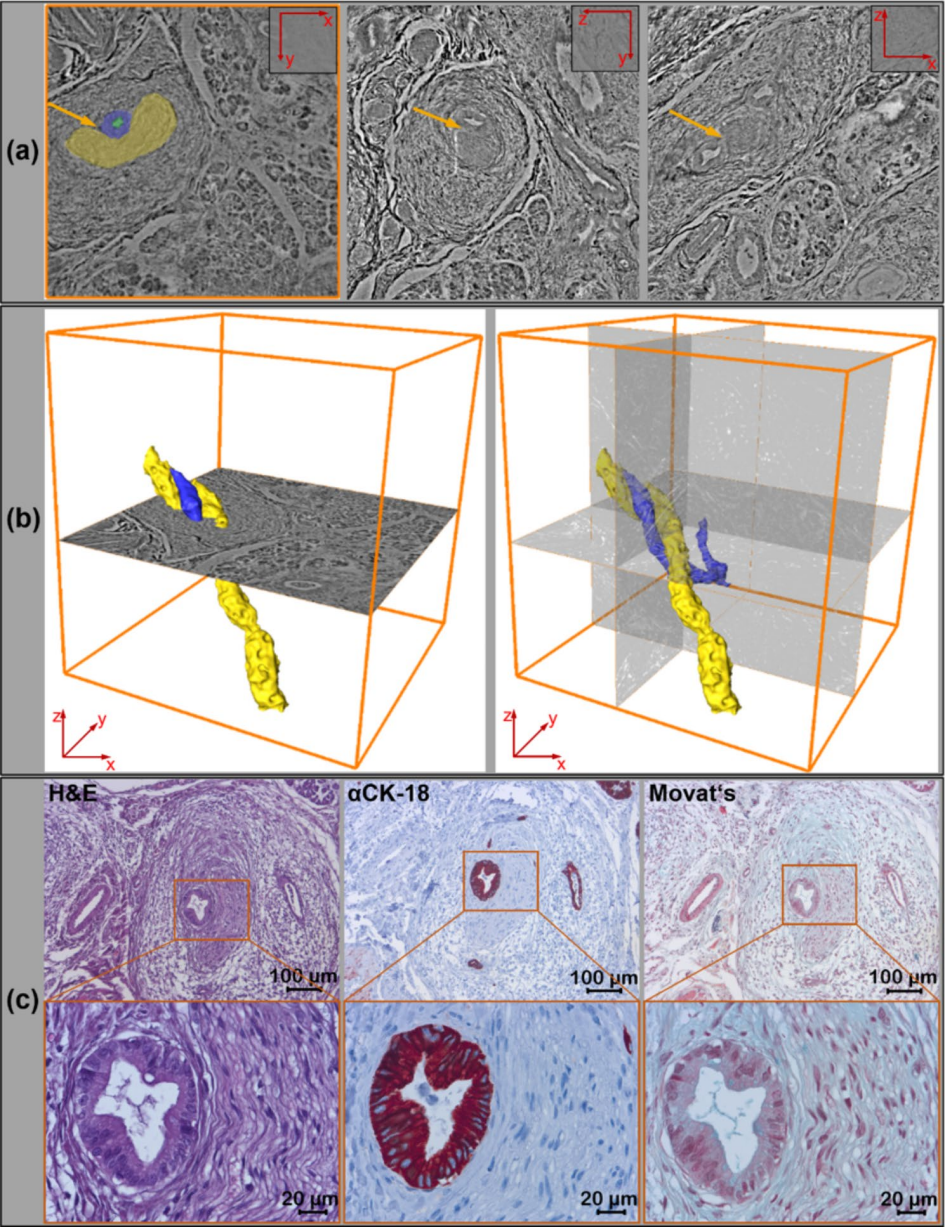
Representative 2D/3D image analysis to assess suspected perineural tumor cell infiltration. Analysis of the X-ray based data set in all 3 spatial directions revealed potential perineural infiltration by a dysplastic pancreatic duct (yellow arrow), as shown in the virtual sections within the punch biopsy (a). Manual segmentation of nerve (yellow), ductal epithelium (blue), and ductal lumen (green) was performed using virtual sections within the 3D data set (a, left figure). Red arrows in (a) indicate the displayed spatial direction of the virtual 2D tissue section. Spatial representation of the segmented structures within this punch biopsy showed how the dysplastic pancreatic duct and nerve wrapped around each other (b). Subsequent H&E staining on 2D tissue sections (c, left panel), specific immunostaining of the duct epithelium with anti CK-18 antibody (c, middle panel), and visualization of different cellular and extracellular tissue components using Movat’s pentachrome stain (c, right panel) validated the close localization of the perineurium and pancreatic duct

One of the X-ray data set of a PDAC specimen, taken from a duodenopancreatectomy with R1-status showed a duct with epithelial proliferation and partially papillary architecture located in close proximity to a peripheral nerve. To address the question of whether perineural infiltration was present in the biopsies the tomographic data set was first evaluated in three planes (Fig. 5a). The collagen fibers of the perineural sheath displayed a higher contrast than the nerve fibers per se, and the epithelium of the pancreatic duct and its lumen were clearly visible. In the virtual section planes epithelial cell infiltration of the perineurium was clearly visualized (Fig. 5a, arrow). Therefore, in a second step, both structures, the pancreatic duct and the adjacent peripheral nerve were segmented semi-automatically and stained virtually. Figure 5b shows the spatial topology of the two structures with their connection and intersection. Although the fusion of the duct with nerve tissue was not clearly detectable, the spatial proximity of these structures suggests disintegration of the perineurium. Combining these findings with histological and immunohistochemical results using Movat’s pentachrome stain marking collagenous structures, and anti CK18 stain depicting epithelial cells, supports the assumption of a present perineural infiltration (Fig. 5c).

## Discussion

With the present study, we show a 3D virtual histology approach of paraffin-embedded unlabeled PDAC biopsies using X-ray based data sets to achieve a detailed representation of relevant pancreatic cancer characteristics. The biopsy can thus be analyzed at the cellular level, with a voxel size of 1.3 μm in tomographic slice images or evaluated as a 3D volume with assessment of the precise distribution of pancreatic ducts, vessels, nerves and desmoplastic tumor stroma. This provides a comprehensive picture of pathologic changes in these individual structures and tissue components. The epithelial growth patterns, for example, can be locally resolved and complex intratumoral events such as perineural/venous/lymphatic invasions can be assessed in their entirety. With this approach, imaging and staging of PanIN and IPMN in a tumor volume have the potential to contribute to diagnosis. This initial 3D overview of diagnostic tissue biopsies is helpful to not miss the presence of PanIN and IPMN as these common precursor lesions of PDAC determine the diagnosis including staging, therapy and prognosis [15, 22, 44]. Furthermore, we show, that the epithelial height of pancreatic ducts as critical feature of the PanIN staging varies enormously within the distance of one tracked pancreatic duct. The comprehensive knowledge of ductal morphology and its epithelial stratification are key features for PanIN grading and has been shown to influence therapeutic decisions [9–11]. The method presented here for tomographic and 3D volume imaging of pancreatic tissue by PCCT is thereby excellently suited to identify characteristic features of PanIN lesions of different grade. Furthermore, with the detailed identification of the resection margins in 3D and arbitrary planes of the scanned tissue specimen the R-status can be determined comprehensively. As a known pivotal parameter of prognosis and diagnosis, the R-status influences postoperative treatment [17, 18]. Therefore, PCCT provides a valuable, comprehensive contribution to a detailed diagnosis. That phase-contrast X-ray tomography has additional, valuable 3D information on the spatial course and accurate assessment of resection margins in cancer surgery has already been shown by Twengström et al. [45]. While the finding of PanIN-1 and PanIN-2 in pancreas biopsies is commonly not an indication for therapeutic interventions, surgical resection is recommended for PanIN-3 lesions [44]. Despite decades of intensive research, PDAC subclassifications based on morphological features or gene mutations have so far not had a significant impact on the therapeutic approach [46, 47]. However, an accurate histopathological examination of PDAC specimens for differential diagnosis still provides a good prognostic assessment [14, 22]. The presented approach of an additional X-ray based data set of the tumor biopsy at microscopic resolution offers a comprehensive examination of the entire tumor material and is a valuable adjunct prior to histological workup of tumor specimens. One of the reasons why PDAC so often has a fatal outcome is its rapid invasion and metastasis [12, 14]. Perineural invasion has also been considered as a hallmark of particularly aggressive tumors [48]. In this study, we demonstrate that peripheral nerves and thus perineural invasion can be tracked and evaluated in the entire biopsy sample volume. Invasion into vessels and nerves is a critical requirement for distant metastasis and relapse of PDAC. At the time of diagnosis, most PDAC patients already have metastases, also independent of tumor size [49, 50]. Tumor invasion into small caliber veins is another typical feature of PDAC and directly associated with the occurrence of liver metastases and poor prognosis [51–53]. The incidence of perineural invasion in PDAC tumors has been reported at 98% with several clinical implications: (i) the occurrence of perineural invasion is a marker for the metastatic potential of PDAC and is associated with increased lymph node metastasis [48, 54]. (ii) It predicts a decreased overall survival, often independent of staging [55, 56] and (iii) is the main cause of tumor associated pain [57]. A number of authors have suggested an 100% occurrence of perineural invasion in PDAC, if only a sufficient number of histological sections were analysed [55, 58, 59]. Finding perineural invasion in a tissue volume, as demonstrated by the X-ray-based method described here, proves that a given pancreatic neoplasm is malignant and no longer a precursor lesion and has therefore crucial consequence for diagnosis and treatment. There are efforts to develop therapeutic approaches that can treat and prevent perineural invasion and thus metastasis at an early stage of PDAC [60]. To implement X-ray based tumor analysis in standard pathology in the future, a validation step by performing conventional histology and immunohistochemistry on paraffin sections obtained from the scanned punch biopsies is required. We have demonstrated not only the correctness of the findings determined by X-ray based data sets, but also that subsequent histological and immunohistochemical examinations are possible at any time after X-ray based tomography. Thus: (i) the 3D findings can be expanded including established diagnostic tumor markers like CK-18 protein. (ii) Histological analysis applied after the X-ray scan validates the morphological features of the volume data set, and (iii) the iterative comparison of 3D information with 2D histological information from the same tumor sample will define and evaluate the diagnostic value of new 3D imaging markers for PDAC. Here we show that the X-ray based data sets of PDAC precursor lesions were visualized as extended, multiform, contiguous 3D structures that can only be seen as planar ductal extensions in 2D microscopy. So far, the morphology of precursor lesions and PDAC subtypes was described two-dimensionally in classical diagnostics. It has been shown that a 2D diagnosis of cancer underestimates the true complexity of tumor growth and progression [26]. Alternative approaches to assess diagnostic features of PDAC and its precursors as well as tumor heterogeneity in 3D are the stacking of sequential immunohistochemically stained tissue slices, which is a very time consuming method [26, 61]. The advantage of this approach is the free rotatability of the structures of interest in 3D and the very detailed overview of the cancer-tissue interface with antibody labeling of the tumor cells [26]. A significant drawback of the use of histological tissue stacks is the irreversible specific staining of all tumor sections and the destruction of the tissue sample as entirety. Fluorescence-based light sheet microscopy (LSFM) has recently also been used to visualize true volume data and tomography and allows the assessment of the mass and distribution of endocrine islets of Langerhans in PDAC, the diabetic pancreas and venous invasion in pancreatic cancer [62–64]. In contrast to the X-ray approach with native paraffinized tissue samples, a complex clearing procedure and adjustment of the refractive index precedes the use of this technique [65]. By using fluorescence-based immunolabeling, the 3D composition of the neoplastic pancreatic tissue with specifically labeled immune cells, vessels, and nerves could be differentially visualized [66]. Compared to the time-consuming preparation steps of LSFM with clearing and additive immunolabeling, scanning native paraffinized tumor tissue as used in this study is fast and straightforward and allows the identification of cell types based on their morphological features [64, 66]. We have shown here that paraffinized tissue blocks do not require specific contrast agent-based contrasting to unambiguously assign morphological structures, as was still necessary at the beginning of X-ray based PDAC histology and is suitable for small punch biopsies to entire paraffin blocks [33]. Although the investigations shown here are a first proof of concept study with only a small sample size, the results provide a promising technical approach to gain important knowledge for PDAC diagnosis. The presented image quality and scan time at the synchrotron setup would allow a broad clinical application of the PCCT approach. To make this accessible to a wider range of pathologists, we propose a two-winged approach: First, because of the short measurement times and high degree of automation possible with synchrotron radiation and specialized radiation equipment, high-throughput biopsy analysis seems feasible in a reasonable time frame. A rapid access scheme and further standardization would be required. Here, fully automated scans, reconstructions and analysis in high throughput is the major challenge for future work. Second, laboratory instrumentation and analysis pathways could be further improved and scaled towards histopathological applications. An obvious concern is the sample transfer from the clinic to the synchrotron. The example of the SYRMEP beamline at the Elettra synchrotron in Trieste, Italy shows that at least for synchrotrons in close proximity, clinical use of synchrotron radiation for 3d histology is possible [67]. Typical scan times for a biopsy take several hours, but a dedicated instrument could be installed directly at the pathologist’s site. Here, machine learning-based segmentation and interpretation could possibly compensate for the inferior image quality compared to synchrotron radiation.

## Conclusion

In summary, we show here, that the 3D X-ray based data sets of PDAC biopsies is a valuable approach to determine the 3D volume morphologies of altered pancreatic ducts including altered stratification of the epithelium and the important diagnostic feature of perineural infiltration. These 3D imaging data have the potential to add comprehensive information and may contribute to a refined diagnosis and improved prognostic and therapeutic patient stratification.

## Data Availability

The data are available from the corresponding author upon reasonable request.

## Abbreviations

AEC: 3-Amino-9-ethylcarbazole
BMBF: Bundesministerium für Bildung und Forschung/German Federal Ministry of Education and Research
CK-18: Cytokeratin 18
DFG: Deutsche Forschungsgemeinschaft/German Research Foundation
H&E: hematoxylin and eosin
IPMN: Intraductal mucinous neoplasia
LSFM: Light sheet microscopy
MBExC: Multiscale Bioimaging:from Molecular Machines to Networks of Excitable Cells
PanIN: Pancreatic intraepithelial neoplasia
PCCT: Phase-contrast X-ray computed tomography
PDAC: Pancreatic ductal adenocarcinoma
PTA: Phosphotungstic acid
3D: Three-dimensional
2D: Two-dimensional
